# Sense of Personal Control Intensifies Moral Judgments of Others’ Actions

**DOI:** 10.3389/fpsyg.2019.02261

**Published:** 2019-10-04

**Authors:** James F. M. Cornwell, E. Tory Higgins

**Affiliations:** ^1^Department of Behavioral Sciences and Leadership, United States Military Academy, West Point, NY, United States; ^2^Department of Psychology, Columbia University, New York, NY, United States

**Keywords:** morality, sense of control, intuition, regulatory focus, judgment, misattribution

## Abstract

Recent research in moral psychology has highlighted how the current internal states of observers can influence their moral judgments of others’ actions. In this article, we argue that an important internal state that serves such a function is the sense of control one has over one’s own actions. Across four studies, we show that an individual’s *own* current sense of control is positively associated with the intensity of moral judgments of the actions of *others*. We also show that this effect extends not only to judgments of rightness and wrongness (Study 1), but also to assignments of reward and punishment (Study 2). Finally, we demonstrate that this effect is based on the current experience of control by showing a moderation of the effect via motivational states (promotion; prevention) that either lead one to incorporate or disregard internal states when making judgments (Study 3) and by subtly manipulating participants’ sense of control (Study 4).

## Introduction

Perceptions of the control that others have over their actions are widely acknowledged to be relevant to moral judgments (Wegner, 2003; Nichols and Knobe, 2007; Pizarro and Helzer, 2010; Sarkissian et al., 2010). When making judgments of others, individuals take into account the degree to which moral actors *intend* to perform the actions being judged (Pizarro et al., 2003a) and the degree to which they can be held *responsible* for performing those actions (Gray and Wegner, 2009). Similarly, when individuals are made to believe that the world is deterministic, they are more likely to engage in immoral behavior, presumably because they absolve themselves of responsibility for their actions (Vohs and Schooler, 2008). When making moral judgments, individuals appear to take into consideration the perceived *responsibility* that an actor has regarding his or her action.

What these studies have in common is that they investigate the degree to which the perceived control of the *actor* over his or her action influences observers’ judgments of that actor’s action. Indeed, recent decades have seen an explosion of interest in the variety of elements that contribute to our judgments of the morality of the actions of others. However, whereas researchers of moral judgment have been interested traditionally in the behaviors, actors, and situations being judged, more recent research has focused on how the qualities and characteristics of the *observer* affect moral judgments (Miller and Cushman, 2013). Such research has included, but is not limited to, political differences (Graham et al., 2009), differences in emotional sensitivity (Schnall et al., 2008; Eskine et al., 2011), and differences in motivational orientation (Janoff-Bulman et al., 2008; Cornwell and Higgins, 2013). In this paper, we argue that another individual difference contributes to the moral judgment process: differences in one’s current sense of control over one’s *own* actions.

There is evidence in the literature which suggests that increasing one’s sense of personal control would result in more intense judgments of others. Research on power (a domain related to sense of control) has shown that those who feel powerful engage in more moralizing reasoning (Lammers et al., 2010) and are motivated to overcome ambiguity in assigning moral traits to individuals (Chen et al., 2004). Given that power is related to a sense of *control* over the world (Galinsky et al., 2003), we might expect a greater sense of control to be associated with stricter moral judgments of others. But why?

Investigations into the role of perceived control in social judgments have thus far tied into the study of attributions. Research has shown that individuals are motivated to explain the actions of others (Kelley, 1973). One of the ways in which they do this is to explain others’ actions in terms of a combination of dispositional characteristics of the actor and contextual factors surrounding the action (Kelley and Michela, 1980). Moral judgments of the actions of others also require explanation in order to assign blameworthiness or praiseworthiness to the actions. There has been a great deal of research examining the complexity of factors that goes into judgments of blame and praise of others (Pizarro et al., 2003b), but, generally speaking, research has explored how attribution of behaviors to contextual factors for behavior leads to less intense moral judgments of others (Alicke, 2000). For the most part, the research on this subject has examined immoral actions, but there is evidence that an obvious lack of intention in doing good can attenuate moral praise as well (Knobe, 2003).

Several theories have been advanced to explain individual differences in the tendency to focus on dispositional versus contextual factors in explaining the behavior of others. Research has shown such differences can arise due to simple self versus other differences in perception (Storms, 1973) or to differences in knowledge about selves versus others (Nisbett et al., 1973). Differences can also arise from cultural norms that highlight dispositional factors or contextual factors when rendering social judgments (Morris and Peng, 1994).

Like attributions, individual differences in sense of control have been the subject of a good deal of study (Phares et al., 1971; Pittman and Pittman, 1980). Generally speaking, the sense of control one has is the degree to which an individual believes one’s actions to be caused by oneself and not by outside factors. In other words, the more one perceives one’s actions and effects in the world to be sourced in oneself, the greater the sense of control one has and vice versa (Vallacher and Wegner, 1989). This also highlights the importance of the attribution process for oneself, and different motivations have been found to underlie differences in explanations for one’s own successes and failures as being caused by oneself or outside factors (Basgall and Snyder, 1988; Sechrist et al., 2004). Importantly for our purposes, and for the purposes of research in moral judgments of others, the sense of control has been shown to be implicated in the willingness to provide contextual judgments for socially relevant events in the world. For example, research has shown that those with a lower sense of control (as a result of their lower subjective socio-economic status) are more willing to attribute things like the rise in income disparities in society as being due to contextual factors beyond the control of the individuals involved (Kraus et al., 2009). Other research on locus of control has shown that those with a more internal locus of control provide harsher judgments under certain contexts (Cherry and Fraedrich, 2000).

Given this research, we should expect that those who have a lower sense of control would be more willing to provide less morally intense judgments of others’ actions, with the opposite being true of those with a high sense of control. However, the research of Kraus et al. (2009) speculated that the association between a preference for contextual factors and global low sense of control is due to the practice of using contextual factors in one’s own life to explain one’s own outcomes. In contrast we argue that recent research on moral judgment suggests that an individual’s perceived sense of control in the moment could increase or decrease moral judgment intensity directly.

Traditionally speaking, moral judgment is supposed to be based upon a consideration of the factors of an actor’s character and situation. From this perspective, human beings deliberately consider various factors that go into a particular behavior in a situation, and render their judgment based on those factors. Research has confirmed that many of these factors are, in fact, used in the formulation of moral judgments. For example, the consequences of behaviors matter (Greene, 2008), as do the perceived desires of the individual actor (Pizarro et al., 2003b). More recent research has demonstrated that the idea of individuals rationally rendering judgments is perhaps overblown (Haidt, 2001), but even those who favor an intuitionist framework ground those intuitions in cultural experiences of the world and people’s roles in it (Haidt and Joseph, 2004, 2007).

More recent considerations of moral psychology suggests that individuals do not simply render judgments based on their perceptions of the person and situation, but instead on their own internal experiences while considering the behaviors in question. For example, research has shown that disgust sensitivity – the degree to which an individual is prone to experiencing disgust – can serve to intensify judgments of others (Horberg et al., 2009). Some researchers have suggested that individuals render judgments, in part, by putting themselves in the place of the actor, and judging how performing the action in question would make them feel (Miller and Cushman, 2013). Thus, those who find the behavior more aversive to perform themselves would provide a more negative moral judgment when that behavior is enacted by others. There is a good deal of empirical evidence that is consistent with this interpretation, including judgments that behaviors that are simply aversive to perform are considered wrong, in spite of their taking place within contexts in which no negative outcomes will result from their taking place (Cushman et al., 2012).

This research suggests that individuals may implicitly use themselves as models for personal responsibility for moral actions during moral judgments. In other words, one’s own sense of control becomes additional information for judging others’ moral worth by providing information concerning how responsible people are for their behaviors more generally. Behaviors would be considered more right or wrong if the world is the sort of place in which people engage in their actions willfully, and one’s internal sense of control could provide information concerning whether or not the world has that characteristic (if their actions feel willful and controlled) or not (if their actions feel random and uncontrolled). Another way to think about this is that one’s own feelings of control provide information concerning whether contextual or personal attributions for behaviors are more appropriate while considering the actions of others.

The present research considers this possibility. We predicted that an individual’s own sense of control would be positively related to the intensity of their moral judgments of others’ actions (Study 1). Furthermore, if these shifts in judgment intensity are the result of shifts concerning whether actions are more or less willed by those being judged, then this intensity should also translate into a willingness to apply greater punishments for bad behavior and provide greater rewards for good behavior (Study 2). Additionally, if these judgments are due to in-the-moment consideration of one’s internal states when making judgments, then manipulating an individual’s willingness to consider internal cues should influence the connection between the sense of control and judgment intensity (Study 3). One subtle way to do so is via regulatory focus: Research has shown that those in a promotion state are more likely to incorporate internal intuitions into moral judgments compared to those in a prevention state (Cornwell and Higgins, 2016). This would also show that the association between sense of control and moral judgment intensity was not simply caused by beliefs about the world. Finally, if individuals are directly considering their internal states and not their beliefs about the world more generally, then if they are put into a momentary state in which they feel as though their control over their own actions is increased or reduced, the intensity of their moral judgments of others should be influenced accordingly (Study 4). We tested these hypotheses in four studies.

## Study 1

All of the above hypotheses rely on the basic premise that a higher self-reported sense of control over one’s own actions will be positively correlated with the intensity of moral judgments of others. We test this basic prediction in Study 1.

### Materials and Methods

#### Participants

Seventy-nine participants were recruited from the subject pool provided by Amazon’s Mechanical Turk for the sum of one dollar. The sample consisted of 40 males and 39 females with a mean age of 35.35 years. There were no significant sex differences for either of the variables examined in this study. We were uncertain what the effect size of the relation would be, so we aimed for a total sample of approximately 80 participants to allow for sufficient power (0.80) to detect a moderately strong (0.30) correlation. To ensure English language proficiency, we limited our sample to the United States. Given cross-cultural differences in morality, this may limit the generalizability of our effect, but we believed that ensuring understanding of the materials was a priority.

#### Procedure

Participants were first presented with a series of fourteen morally charged scenarios inspired by a variety of research paradigms in the moral judgment literature. The scenarios included morally dilemmatic vignettes (e.g., an adaptation of the Heinz dilemma from Kohlberg, 1969), clearly moral actions (e.g., keeping one’s promises even when there are more pleasurable alternatives available), and clearly immoral actions (e.g., cheating on an exam). We also included some scenarios inspired by Haidt et al. (1993) that involved immoral acts typically associated with moral dumbfounding (Haidt, 2001). While these different scenarios have been used to different ends in past research (e.g., Kohlberg’s Heinz dilemma was used to assess moral reasoning; Haidt’s social intuitionist scenarios for demonstrating the breadth of moral concern), what we were interested in was the degree of *intensity* of the judgment once rendered, and had no reason to suspect that the effect would be limited to a particular kind of moral scenario. Therefore, we wanted to test whether the effect would obtain for as wide a variety of scenarios as possible. The full scenarios are available in Appendix A, and the results for each scenario are reported in Table 1.

**TABLE 1:** Correlations of moral judgment intensity with sense of control for each scenario across four studies.

**Scenario**	**Study 1**	**Study 2**	**Study 3^a^**	**Study 4^b^**
1	0.14	0.17	0.35^∗∗^(0.16)	0.02
2	0.21	0.25^∗∗^	0.56^***^(0.27^∗^)	0.03
3	0.14	0.15	0.31^∗^(0.10)	0.05
4	0.31^∗∗^	0.21^∗^	0.48^*⁣**^(0.32^∗∗^)	0.12
5	0.14	0.18	0.48^*⁣**^(0.29^∗^)	0.00
6^c^	0.12	0.29^∗∗^	0.60^*⁣**^(0.42^*⁣**^)	0.02
7	0.23^∗^	0.30^∗∗^	0.56^*⁣**^(0.55^*⁣**^)	0.11
8^c^	0.13	0.09	0.60^*⁣**^(0.31^∗^)	0.06
9^c^	0.15	0.18^∗^	0.34^∗∗^(0.20)	0.08
10^c^	0.07	0.22^∗^	0.46^*⁣**^(0.26^∗^)	0.06
11^c^	0.06	0.14	0.54^*⁣**^(0.22)	0.12
12	0.21	0.26^∗∗^	0.46^*⁣**^(0.55^*⁣**^)	0.24^∗^
13	0.21	0.17	0.27^∗^(0.43^*⁣**^)	0.14
14	0.29^∗^	0.19^∗^	0.44^*⁣**^(0.27^∗^)	0.24^∗∗^
All right	0.37^∗∗∗^	0.33^∗∗∗^	0.62^*⁣**^(0.37^∗∗^)	0.18^∗^
All wrong	0.17	0.25^∗∗^	0.61^*⁣**^(0.48^*⁣**^)	0.10

**^∗^p < 0.05; ^∗∗^p < 0.01; ^∗∗∗^p < 0.001. ^*a*^Study 3 shows the correlation in the promotion condition with the correlation in the prevention condition in parentheses. ^*b*^Study 4 examines the effect of the manipulation (0 = Random condition; 1 = Choice condition). ^*c*^Denotes scenarios generally considered more morally wrong than right; the remainder are generally considered more right than wrong.**

For each scenario, participants were asked to make three judgments on 9-point scales: whether the behavior of the actor was morally right or wrong from 1 (completely morally wrong) to 9 (completely morally right), how morally wrong the behavior is from 1 (not at all morally wrong) to 9 (extremely morally wrong), and how morally right the behavior is from 1 (not at all morally right) to 9 (exceptionally morally right). These were all highly correlated with one another (general morality – moral rightness: *r* = 0.96, *p* < 0.001; general morality – moral wrongness: *r* = 0.78, *p* < 0.001; moral rightness – moral wrongness: *r* = 0.78, *p* < 0.001). By including both the rightness and wrongness judgment opportunities, participants would not feel as though there was a normative judgment for the scenarios, particular those that were more morally ambiguous (e.g., the Heinz dilemma). However, since the first judgment was on the overall morality of the action, we used it to compute judgment intensity. The ‘intensity’ of the moral judgment was computed by calculating the absolute value of the distance from the center value of the scale (in this case, 5). That is, more intense judgments made greater use of the outer scale points relative to the inner points. For this study, the order of the scenarios was randomly generated, and then each participant viewed them in that fixed order.

Following the scenarios, participants indicated their sense of control. Sense of control was calculated using a scale measuring participants’ sense of willfulness and deliberateness of their actions in the experiment (Wegner et al., 2004; or see the Appendix in Cornwell and Krantz, 2014). This scale consists of six items, in the following order: “How much control did you feel in the task?” “To what extent did you feel your actions to be deliberate?” “To what degree did you feel that the judgments belonged to you?” “To what degree did you feel you were responsible in this task?” “To what extent did your judgments feel voluntary?” “To what extent did you feel willful?” These items were all rated on a scale from 1 (not at all) to 9 (very much so). An average of the scores on each of these items represented the participant’s sense of control score.

#### Analysis

Because individuals differ in their overall willingness to provide intense or non-intense judgments for a variety of reasons unrelated to our hypotheses, and because certain scenarios (such as the dog-eating or incest scenario) elicited far more extreme judgments than others (such as the Heinz dilemma), a crossed-random effects model was used to analyze the data. This model allowed us to include both individual differences and scenario differences in the model as random effects, rather than simply taking the mean level of intensity across such varied scenarios. Furthermore, because there is no straightforward way to estimate effect sizes in these models, we report the effect size of a simple regression of the mean level of intensity onto personal sense of control in footnotes with the primary effect of interest in each study.

### Results and Discussion

The sense of control scale (*M* = 7.66, *SD* = 1.42) had high internal reliability (α = 0.88). Consistent with our predictions, self-reported sense of control was significantly positively associated with moral judgment intensity (*M* = 2.83, *SD* = 0.66) across the judgments in this study (*b* = 0.16, *SE* = 0.05, *z* = 3.15, *p* = 0.002, 95% CI = [0.060, 0.259])^1^. Thus, our primary hypothesis was confirmed. The effect for each scenario, and the scenarios categorized as positive or negative, can be found in Table 1 for this and all subsequent studies.

This study provided preliminary evidence that individuals who experience a greater sense of personal control also provide more intense moral judgments of others. This is consistent with the findings of Kraus et al. (2009) extended to the domain of moral judgment. The purpose of Study 2 was to replicate this finding while also extending it by showing that this intensity also applies to a willingness to impose harsher punishments for wrongdoing and provide larger rewards for good deeds compared to those experiencing less personal control. Furthermore, in Study 1 the sense of control judgments followed the moral judgments, and the causality could therefore run in the opposite direction. Therefore, in Study 2, we had participants complete the sense of control judgment prior to judging these 14 scenarios.

## Study 2

In this study, participants were given the opportunity to either assign a punishment or a reward for a given action. Following this component, participants reported their sense of control, and then judged the same 14 scenarios used in the previous study. This latter portion would serve as a replication of our finding in Study 1.

### Materials and Methods

#### Participants

Since in this study we would be averaging the amount of redress from two different kinds of situations, we expected greater variability and, therefore, a smaller effect size for one of the outcome variables. Thus, we increased our sample size, aiming for approximately 120 participants. One hundred fifteen participants were recruited to complete the study via Mechanical Turk for the sum of one dollar. Participants consisted of 53 males and 62 females with a mean age of 36.63 years. There were no significant sex differences in this study. Participants were randomly assigned to the two conditions described below. Once again, to ensure English language proficiency, we limited our sample to the United States.

#### Procedure

The procedure for this study was identical to Study 1 except that the sense of control questionnaire and the moral scenarios were preceded by either a judgment involving the assignment of a punishment for a crime committed or a reward for community service hours completed. The punishment scenario was worded as follows:

Imagine that you are a judge presiding over juvenile delinquency proceedings. A seventeen-year-old male was convicted of spray-painted offensive words on the outside wall of the local high school, where they could be seen by students at the neighboring elementary school. The specific crime committed is vandalism, and as the judge, it is your job to assess his penalty for this act.

Legal statute states that such behavior is punishable by mandatory community service of no fewer than 5 h and no more than 35 h. Typically, comparable offenses in the past usually received sentences of 20 h from other judges.

Below, please indicate the number of community service hours the young man should receive for his offense.

The reward scenario was worded as follows:

Imagine that you are a judge presiding over scholarship award proceedings. A 17 years old male is to be commended for exceptional service to his community by receiving a scholarship that is given annually. Specifically, he is being recognized for completing an exceptional number of service hours, and as the judge, it is your job to assess the amount of scholarship money he is to receive for his college expenses.

The guidelines of the scholarship require that students receive at least $2500 and at most $8500. Typically, students with a comparable number of hours have received $5000.

Below, please indicate the amount of scholarship money the student is to receive for his service.

Following the random presentation of one of these scenarios, participants were given the sense of control questionnaire, which they were told to answer with respect to the judgment of punishment or reward that they had just completed, although the wording and ordering of the items were identical to that used in Study 1. Following the sense of control questionnaire, participants were asked to make judgments of the same fourteen scenarios (presented in the same order) from Study 1 that were then analyzed in the same manner described previously.

### Results and Discussion

Once again, the sense of control questionnaire (*M* = 7.34, *SD* = 1.46) showed high internal reliability (α = 0.89). Consistent with the previous study, the greater the sense of control participants reported with respect to their judgments, the greater the scholarship amount they awarded (*M* = 3,612.50, *SD* = 851.32) to the high school student in the positive scenario (β = 0.28, *t*(51) = 2.11, *p* = 0.04, 95% CI = [8.448, 352.655], η^2^ = 0.08), and the greater the amount of punishment (*M* = 31.44, *SD* = 9.01) they applied to the high school student in the negative scenario, though this relationship was not statistically significant, but was in the predicted direction (β = 0.22, *t*(60) = 1.74, *p* = 0.09, 95% CI = [−0.191, 2.36], η^2^ = 0.05). Standardizing and combining these variables into a single “redress” variable (and controlling for the scenario content) yielded an overall significant association between redress and sense of control (β = 0.25, *t*(112) = 2.65, *p* = 0.009, 95% CI = [0.042, 0.290], η^2^ = 0.06).

As in Study 1, there was also a significant association between self-reported sense of control and moral judgment intensity (*M* = 2.69, *SD* = 0.82), such that those who reported higher levels of control also made more intense moral judgments (*b* = 0.19, *SE* = 0.05, *z* = 3.85, *p* < 0.001, 95% CI = [0.093, 0.287])^2^. This was true even when controlling for whether the participant had previously viewed the reward or the punishment scenario, and how much punishment/reward the individual chose to apply in that scenario (*b* = 0.17, *SE* = 0.05, *z* = 3.21, *p* = 0.001, 95% CI = [0.064, 0.266])^3^.

These results replicate our findings from Study 1 and show as well that self-reported sense of control is also associated with the amount of rewards and punishments for positive and negative behaviors individuals are willing to assign. However, although in this study (unlike in Study 1) the sense of control judgments occurred prior to the 14 scenarios, they still took place following a judgment task. Furthermore, since these studies are entirely correlational, it is possible that a third variable, such as beliefs about personal responsibility more generally could be predicting both sense of control and moral judgment intensity. Therefore, in Study 3, we sought to manipulate a variable that should subtly impact participants’ use of their own internal states in forming moral judgments, providing evidence that the effect on judgment intensity flows from those states and not from general beliefs about the world. Sense of control would also be measured prior to performing any sort of judgment task to avoid the possibility of reverse-causality.

## Study 3

According to regulatory focus theory, individuals pursue goals with either a promotion or a prevention focus (Higgins, 1997). While in a promotion state, individuals regard goals as ideals, hopes, and aspirations. In contrast, while in a prevention state, individuals regard goals as responsibilities, duties, and obligations. Based on this difference, research has shown that those primed with a promotion focus are more likely to make use of feelings in decision making, and those primed with a prevention focus are more likely to make use of reasons rather than feelings in decision making (Pham and Avnet, 2004; Avnet and Higgins, 2006). Furthermore, in the domain of moral judgment specifically, those primed with a promotion focus are more likely to make use of their intuitive feelings in making moral judgments, resulting in more intense judgments compared to those primed with a prevention focus if the wrongness of those judgments depends exclusively on intuitive feelings of right and wrong (Cornwell and Higgins, 2016). It is also the case that Americans tend to be more promotion-focused than prevention-focused (Higgins, 2008); because our samples consisted of Americans, this could be an explanation for the robust nature of the effect across studies.

However, even those who are chronically more promotion-focused can be induced into a prevention state. Therefore, when making judgments, we would expect that those primed with a promotion focus, compared to those primed with a prevention focus, will be more likely to make use of their feelings – in this case, experienced sense of control – when formulating judgments of others. If the manipulation moderates the connection between sense of control and judgment intensity, that would suggest that those internal states are being used by participants in making their judgments directly, and are not the result of beliefs about the world more generally (which would be just as likely to be used by those with a prevention focus, see Cornwell and Higgins, 2016). This study tested this potential moderator.

### Materials and Methods

#### Participants

We aimed at a sample size of approximately 120 participants that would be adequate to detect the hypothesized interaction. One hundred twenty-eight participants were recruited from Amazon’s Mechanical Turk for the sum of $2.00. The sample consisted of 84 females and 44 males. Both chronic promotion and prevention were significantly higher among males (promotion: *M* = 3.64, *SD* = 0.71; prevention: *M* = 3.29, *SD* = 0.99) compared to females (promotion: *M* = 3.38, *SD* = 0.62, *t*(125) = −2.09, *p* = 0.04, 95% CI(diff) = [−0.500, −0.014]; prevention: *M* = 2.96, *SD* = 0.80, *t*(125) = −2.05, *p* = 0.04, 95% CI(diff) = [−0.655, −0.012]), so we controlled for sex differences in the analyses below. The average age of the sample was 32.2. Since, unlike the previous studies, this study relied on participants spending an adequate amount of time on the essay task for the induction to work, we conducted a pilot study to determine the typical length in order to exclude participants that completed the study too quickly. In our pilot study conducted at Columbia University (*N* = 78), participants completed the essay and judgment tasks at an average pace of 15 min with a standard deviation of approximately 10 min. Therefore, we excluded one participant from the analysis that completed the study in under 5 min (i.e., one standard deviation below the mean), resulting in a final sample size of 127. The sample was again limited to those residing in the United States.

#### Procedure

Participants were first randomly assigned to one of two groups: the promotion prime and the prevention prime. Each prime involved writing a series of three short essays describing a set of experiences, which is a well-established method for inducing writers into a particular regulatory focus (Higgins et al., 2001; Freitas and Higgins, 2002). For the promotion focus prime, the following prompts were used:

1. Please think back to the times when you felt like you made progress toward being successful in life.
2. Please think back to the time when compared to most people, you were able to get what you wanted out of life.
3. Please think back to the times when trying to achieve something important to you, you performed as well as you ideally would have liked to.

For the prevention focus prime, the following prompts were used:

1. Please think back to the time when being careful enough has avoided getting you in trouble.
2. Please think back to the times when growing up, you stopped yourself from acting in a way that your parents would have considered objectionable.
3. Please think back to the times when you were careful not to get on your parents’ nerves.

After this task, participants filled out the six sense of control items with respect to how they felt in the situations they recalled (i.e., “please answer the following questions about the situations you just recalled”). Thus, the items were worded and ordered as follows: “How much control did you feel in these situations?” “To what extent do you feel your actions were deliberate?” “To what degree do you feel that your actions belonged to you?” “To what degree to you feel you were responsible for your actions in these situations?” “To what extent did your actions feel voluntary?” “To what extent did you feel willful?” Following the sense of control questions, participants were presented with the same fourteen judgment tasks used in the previous studies presented in a random order as in Study 2.

Finally, following the moral judgment tasks, participants filled out the Regulatory Focus Questionnaire (RFQ; Higgins et al., 2001) to be able to test for chronic effects of regulatory focus, and control for it when testing the effects of the manipulation. The RFQ consists of 11 items that measure chronic promotion focus pride (e.g., “How often have you accomplished things that have gotten you ‘psyched’ to work even harder?”) and chronic prevention focus pride (e.g., “Growing up, would you ever ‘cross the line’ by doing things that your parents would not tolerate?” reverse-scored). These items were presented in a fixed order. Although this measure appeared in the same session as the regulatory focus priming that occurred at the outset of the study, it was sufficiently temporally removed because it appeared after the agency self-report and moral judgment task. Indeed, the promotion versus prevention priming was not significantly related to either chronic promotion or prevention focus pride (*t*s < 1).

### Results and Discussion

As with the previous studies, the sense of control items (*M* = 7.53, *SD* = 1.33) had high internal reliability (α = 0.91). Those in the promotion-primed group reported somewhat higher sense of control (*M* = 7.74, *SD* = 1.25) compared to those in the prevention-primed group (*M* = 7.33, *SD* = 1.39), but this difference was not statistically significant (*t*(125) = −1.75, *p* = 0.08, 95% CI(diff) = [−0.875, 0.053]). There were significant associations, however, between chronic promotion focus pride (*M* = 3.47, *SD* = 0.66) and sense of control (*r* = 0.28, *p* = 0.001) as well as chronic prevention focus pride (*M* = 3.07, *SD* = 0.88) and sense of control (*r* = 0.18, *p* = 0.05). This is unsurprising since research has shown that regulatory focus, particularly the promotion focus, is associated with greater illusions of control (Langens, 2007). Nevertheless, these findings prompted us to control for chronic regulatory focus when testing the manipulation effect in this study and again in Study 4.

**FIGURE 1:**
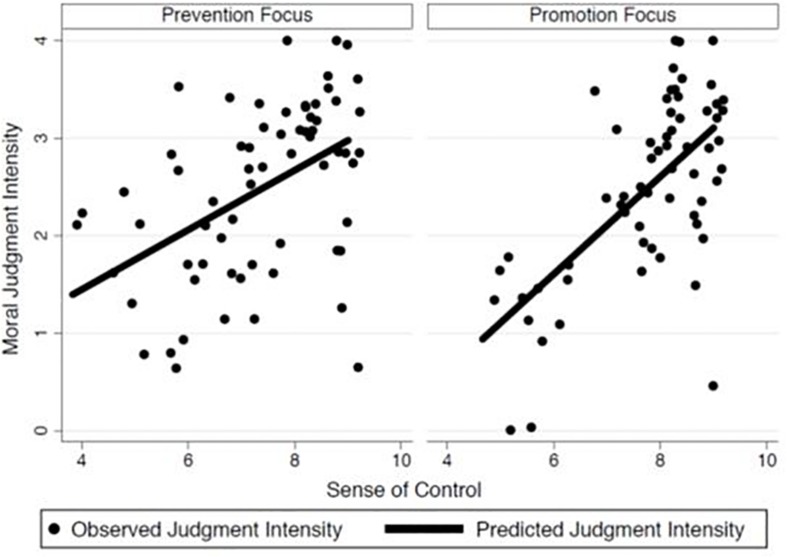
Relation between sense of control and moral judgment intensity by regulatory focus priming condition (Study 3). Points have been “jittered” to prevent stacking.

Controlling for chronic promotion and prevention and sex differences, the association in this study between sense of control and moral judgment intensity was significant (*b* = 0.40, *SE* = 0.05, *z* = 7.61, *p* < 0.001, 95% CI = [0.293, 0.497])^4^. Regarding the main purpose of the study, there was a strong association between sense of control and moral judgment intensity in the promotion condition (*b* = 0.54, *SE* = 0.08, *z* = 7.07, *p* < 0.001, 95% CI = [0.390, 0.690])^5^. There was also a significant, but weaker, association between sense of control and moral judgment intensity in the prevention condition (*b* = 0.30, *SE* = 0.07, *z* = 4.35, *p* < 0.001, 95% CI = [0.162, 0.429])^6^. This difference in differences was strong enough to produce a significant interaction, such that the relation between sense of control and moral judgment intensity was attenuated in the prevention condition compared to the promotion condition (*b* = 0.20, *SE* = 0.10, *z* = 1.99, *p* < 0.05, 95% CI = [0.003, 0.392])^7^. The within-condition effects for induced regulatory focus are shown in Figure 1. Notably, there were no significant differences in moral judgment intensity resulting directly from the type of regulatory focus priming that participants received (*z* < 1).

These results suggest that sense of control does influence assessment of moral and immoral behavior in light of the current feeling states of the observer, specifically the current state relating to feelings of control, given that this effect can be enhanced via a regulatory focus state that increases the extent to which an individual pays attention to his or her internal states when making judgments (i.e., a promotion focus). This suggests that the effect is rooted in the current experiences of the observer, rather than beliefs about the world in general. One remaining question that relates to the central concerns of this investigation is whether these experiences of control can be experimentally induced, and then, as momentary experiences, can influence the intensity of moral judgments. This possibility was tested in Study 4.

## Study 4

Self-reported experience of personal sense of control could be influenced by chronic factors such as having practice making dispositional or contextual attributions. To control for such possible chronic factors, in Study 4 we manipulated the amount of choice participants perceived themselves to have in how the moral judgment task itself proceeded. We then sought to determine whether this manipulation of the sense of control could influence moral judgment intensity in the same way as self-reported sense of control. Specifically, we examined whether participants in a condition where they have the illusion of choice would make more intense moral judgments compared to those in a condition where the order of scenarios was explicitly random.

### Materials and Methods

#### Participants

We aimed for an ultimate sample size of around 100 to power a simple comparison between two groups. However, unlike previous studies, we anticipated attrition in this sample given our manipulation (see below). Therefore, we maintained our target sample size of approximately 120 participants. One hundred nineteen participants from the Behavioral Research Lab at the Columbia Business School participated in this study in exchange for entry into a raffle to win $75.00. The sample included 38 males and 81 females. There were no significant sex differences for any of the variables in the study. Age data was not collected in this sample. Eighteen participants were excluded due to suspicions or concerns voiced about the study when given the opportunity to do so (see below), leaving 35 males and 66 females for the final analysis. There was no significant association between voiced suspicions/concerns and being placed into the choice (*N* = 11) or random (*N* = 7) conditions (*z* = 1.14, *p* = 0.25).

#### Procedure

Participants first filled out a Regulatory Focus Questionnaire (Higgins et al., 2001). Following this questionnaire, participants were informed that they would be randomly assigned to one of two conditions: the “Random” condition or the “Choice” condition. In the “Random” condition, participants were told that they would move from scenario to scenario in an order randomly generated by the computer (as was the case in the previous studies, but now the random process was made explicit). In the “Choice” condition, in contrast, participants were made to feel as though they had control over how the study would progress (although in actuality, their progression would also be random). In this condition, participants were presented with a list of scenarios named Scenario 1 through Scenario 14 and were asked to select which scenario they would like to see first. After selecting a particular numbered scenario and clicking continue, participants were actually presented with a random scenario, regardless of what number they selected. Following their judgment, they were again presented with a list of numbered scenarios, excluding the number that they had previously selected. They again selected a number from the remaining numbers and actually received a random scenario (excluding the scenarios they had received on the prior trials). This process continued until all scenarios were completed. Thus, even though the actual order of presentation of scenarios was random as in the “Random” condition, in this so-called “Choice” condition, participants would feel as though they were in control of which scenario they would receive next – the classic illusion of control.

Finally, because in this study we needed to rely on participants’ belief in the reality of the manipulation, we provided an open-ended space in which participants could report anything odd or suspicious they found about the study. Any participants who reported that they had participated in a study containing the scenarios previously, who guessed the true purpose of the study, or who did not believe the manipulation, were excluded from the analysis.

### Results and Discussion

**FIGURE 2:**
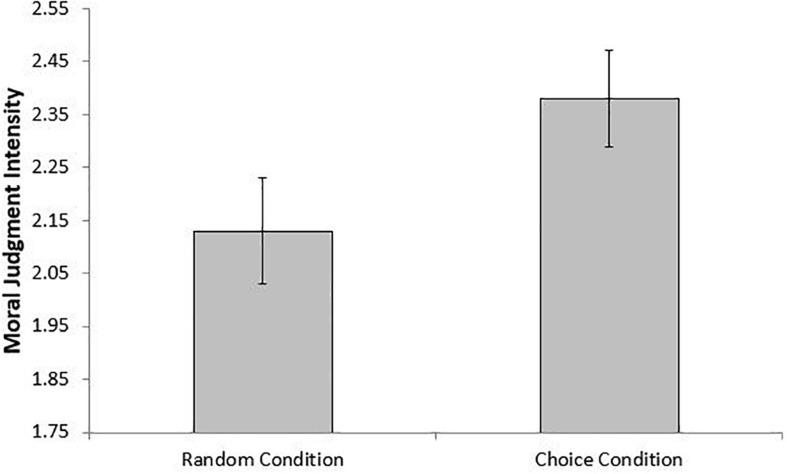
Moral judgment intensity as a function of experimental condition. Bars represent ±1 standard error around the mean (Study 4).

Consistent with our prediction, those in the “Choice” condition reported more intense moral judgments (*M* = 2.41) than those in the “Random” condition, controlling for chronic promotion and prevention focus (*M* = 2.10; *b* = 0.39, *SE* = 0.15, *z* = 2.60, *p* = 0.01, 95% CI = [0.095, 0.683])^8^. This effect is shown in Figure 2. Interestingly, as in Study 3, chronic regulatory focus differences appear relevant to the effect of our manipulation on judgment intensity. Specifically, if chronic promotion and prevention are computed into a difference score such that positive scores indicate a stronger promotion focus and negative scores indicate a stronger prevention focus, the effect of the manipulation is highly significant among those who are more promotion-focused (*b* = 0.45, *SE* = 0.18, *z* = 2.52, *p* = 0.01, 95% CI = [0.101, 0.805]), but not significant among those who are more prevention-focused (*z* < 1). However, unlike the induction in Study 3, this chronic difference in differences was not large enough to significantly moderate the effect of the manipulation on moral judgment intensity, although the effect is in a direction consistent with the findings of Study 3 (*b* = 0.20, *SE* = 0.18, *z* = 1.08, *p* = 0.28, 95% CI = [−0.160, 0.553]).

**FIGURE 3:**
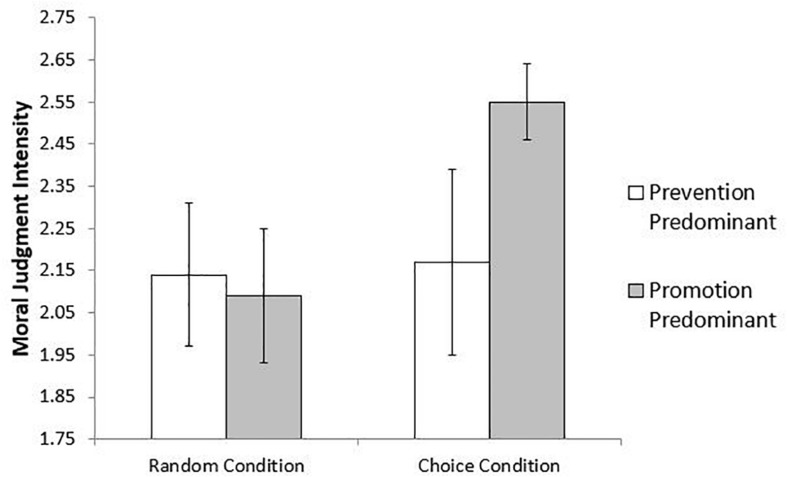
Moral judgment intensity as a function of experimental condition and chronic regulatory focus. Bars represent ±1 standard error around the mean (Study 4).

Interestingly, the effect pattern (shown in Figure 3) suggests that it may be promotion-focused participants in the Choice condition who particularly differ from the other three conditions. Thus, we generated a dummy variable which was equal to 1 if participants had a positive regulatory focus difference score and were manipulated into the Choice condition, and a 0 if they fell into any of the other three groups (i.e., if they had either a negative regulatory focus difference score or were placed into the Random group). The effect of this variable on moral judgment intensity was significant (*b* = 0.43, *SE* = 0.17, *z* = 2.58, *p* = 0.01, 95% CI = [0.103, 0.754)], such that promotion-focused individuals in the Choice condition provided more intense judgments compared to all other participants. These results are again consistent with the association between sense of control and moral judgment intensity, and are consistent with the theory that the effect on moral judgment intensity is rooted in participants’ current internal state.

## General Discussion

Past research has highlighted both the importance of observers perceiving that a target actor is in control of his or her action when they make judgments about that actor, and the importance of observers’ own intuitive internal experiences when they make moral judgments of others’ actions. Bringing these literatures together suggests the possibility that individuals’ experience of their own sense of control can, in the moment, influence the perceived rightness or wrongness of others’ behaviors. The present research, across four studies, supports that there is a reliable positive relation between observers’ sense of personal control and the intensity of their moral judgments of others. Study 2 showed that this effect is not limited to just judgments of rightness and wrongness, but is evident in a willingness to apply punishments and rewards for perceived moral and immoral behavior.

Although these effects are consistent with prior research showing that sense of control is related to different attribution styles, this past research has generally relied on more chronic explanations for this association, such as learning to make such attributions spontaneously through repeated practice or more general beliefs like locus of control. In contrast, Studies 3 and 4 suggest, consistent with recent work in the moral psychology literature, that the impact of an internal experience – in this case, a sense of personal control – on moral judgment intensity can have an effect on judgments in the moment.

By showing a relation between observers’ sense of control while they make moral judgments of others and the intensity of those moral judgments, this research makes an important contribution to the literature on moral judgments, both with respect to theory and societal implications. Our findings show that when we make a moral judgment of another person’s action, we must take into account not only that person’s sense of control over his or her action, but also our *own* current sense of control that could bias our judgment. More research needs to be conducted on this issue, but our results suggest observers’ own sense of personal control makes a significant contribution to our moral judgments of others.

The societal implications for this research depend on the context. On the one hand, it does appear that the more control one feels while making moral judgments, the more intense those judgments will be. This can be beneficial or harmful depending on whether you are on the receiving end of a positive or negative moral judgment of an observer who is currently experiencing personal control over his or her actions. Notably, in either case it is a biased judgment.

There are potentially even broader implications of the present research. For instance, in the domain of morality, researchers have theorized that judgments of good and bad behavior actually involve judging whether individuals have good or bad moral character (see Pizarro and Tannenbaum, 2012). Since character judgments imply a more dispositional attribution style, perhaps this is a factor that is influenced by an individual’s current sense of control, such that the more control one feels the more judgments of others are judgments of character rather than behavior (or the consequences of that behavior). Other research suggests that moral judgments are susceptible to social influence (Kundu and Cummins, 2013). Perhaps these effects are driven by individuals with a low sense of control who are more circumspect in their moral judgments to begin with; that is, perhaps sense of control can act as a potential moderator of social influence on moral judgments.

There is another implication of this research in regards to social status. One might be tempted to infer from our results that individuals who feel less control over their actions (such as those of low subjective socioeconomic status) are less moral people. Instead, our results suggest that they may be less *moralistic* people. Indeed, recent research has suggested that individuals lower on the class hierarchy tend to explain experiences in situational terms, due to their having a lower sense of control (Manstead, 2018). This could lead to individuals lower on the social hierarchy being less morally judgmental of others’ actions, and feeling less morally entitled to whatever beneficial outcomes or rewards they experience. In contrast, those higher in the social hierarchy may be more morally judgmental, and be more likely to see their successes and outcomes as the result of their good behavior. This has led some authors to argue that those higher on the social hierarchy, although more moralistic, may not be more moral, and instead can be more solipsistic in their worldview (Kraus et al., 2012).

It should also be noted that there is research suggesting that those who have fewer material resources are more judgmental when the moral scenarios in question involve harm, and that this effect is mediated by perceived vulnerability (Pitesa and Thau, 2014). This is further complicated by compensatory control theory, which suggests that those who are low in social status would be more moralistic than those high in social status, since clear moral rules provide a means of restoring a sense of control (Kay et al., 2009). Whether those lower or higher in the social hierarchy provide more or less intense moral judgments seems to depend, in part, upon whether they are putting themselves in the shoes of the perpetrator or the shoes of the victim. Future research will need to explore the boundary conditions for these different effects. What our research shows is that observers’ sense of control in the moment intensifies their moral judgments of others.

In addition to these implications, the present research raises a number of theoretical issues. First, although the scales used for our dependent measure were intended to provide indications of judgment intensity, it is also possible that it may be picking up on other factors that correlate with judgments of rightness or wrongness, such blameworthiness, praiseworthiness, or even degree of certainty that the judgment is correct. The measure also fails to distinguish between low intensity borne of uncertainty from low intensity resulting from ambivalence.

Second, an important area of future research involves the search for a more specific mechanism of the effect we highlight here. The studies we report in this paper are more consistent with recent work in moral psychology highlighting the importance of individual differences in the internal states of observers when formulating moral judgments. However, it is unclear how precisely these internal states are implicitly used. Are they used as a way of estimating the sense of control of those engaging in moral and immoral behaviors in order to improve the accuracy of target judgments? Alternatively, are those who experience a greater sense of control over their actions experience stronger engagement when judging others, with stronger engagement intensifying both positive and negative judgments (Higgins, 2006)? Our studies allow us to conclude that the sense of control is directly implicated in moral judgments, is rooted in the observer’s inner states, and is not merely a chronic phenomenon resulting from cumulative experience or beliefs about the world. However, more research is needed to draw conclusions concerning the precise mechanism or mechanisms by which this happens. Once those mechanisms are uncovered, other research will need to explore the degree to which individual differences may moderate the effects we found in a manner similar to regulatory focus moderation.

There are a number of methodological limitations to this research that need to be highlighted as well. As noted above, the main dependent measures are simple vignettes, a few of which are unlikely to be encountered in everyday life. Furthermore, while the main effects were robust and reliable, in some cases they were also quite small. Certainly these small differences might tip the balance, but it is important not to overstate them, particularly given the limited sample size in one of the studies (*N* < 100). An additional criticism of the above studies is that none of them involved having “skin in the game;” that is, none involved following through on moral judgments, but merely extended to hypothetical behavioral intentions (Study 2). This is an issue with the moral psychology literature more generally (see, e.g., the critique in Haidt, 2001), and we hope to have the opportunity to extend this and other research into a more concrete behavioral domain.

Another limitation is the proximity of the independent and dependent measures in the first three studies. Studies 1 and 2, in particular, cannot rule out the possibility that more intense judgments lead to a greater sense of control in participants and vice versa. In addition, it is possible that, at least in part, the association arising between observers’ sense of control and moral judgment intensity could be arising as a result of a third unmeasured variable. For example, there could be a characteristic of the participants that simply leads them to give extreme responses to all survey questions that has nothing to do with the link we hypothesize. The change in design for Study 3 with regulatory focus as an experimentally manipulated moderator addresses the first concern, and the actual manipulation of current sense of control in Study 4 suggests that any potential third variable is unable to explain the hypothesized connection in its entirety. Nonetheless, it is possible that other variables are also at work in these studies. It is important that future research address these shortcomings through replication and extension to additional paradigms that consider other potential mechanisms.

A related limitation is the lack of widespread diversity in the samples. While the Mechanical Turk samples are likely to be more diverse than the university-centered samples, our samples were limited to the United States in order to ensure English proficiency. The diversity of moral reasoning and judgment across cultures is well-documented (e.g., Shweder et al., 1997), and thus future research should widen the scope of sample sources beyond the samples in our studies. In addition, while Study 4 did use a university sample, the first three studies were all conducted on samples drawn from Mechanical Turk. University students and Mechanical Turk workers are likely disproportionately middle and upper class, and, given the effects of socioeconomic status cited earlier, it might be useful to conduct these same studies in populations that cross socioeconomic lines. In brief, in order to ensure that the results are generalizable, future researchers will need to replicate them using other populations. The existence of the moderator of regulatory focus in Studies 3 and 4 in particular highlights the contingent nature of the effects given that there are cross-cultural differences in regulatory focus (Higgins, 2008), and other individual differences that vary by culture and circumstance could potentially moderate the effects.

This research is intriguing, but it is only a starting point. It suggests a link between observers’ experience of control and the intensity of moral judgments of others. However, more research needs to be conducted on the subject to understand its relation to existing constructs on the one hand, such as attribution processes, and its societal implications on the other. That said, this research does contribute additional support to the idea that when working to understand the underpinnings of moral judgment, the experience of the observer must be considered. Specifically, when an observer makes a moral judgment of another person, not only do the circumstances and the nature of that person contribute to the intensity of the judgment, but also the observer’s own internal sense of control.

## Data Availability Statement

The datasets generated for this study are available on request to the corresponding author.

## Ethics Statement

These studies were carried out in accordance with the recommendations of Columbia University’s Institutional Review Board with electronic or written informed consent from all subjects. All subjects gave written or electronic informed consent in accordance with the Declaration of Helsinki. The protocol was approved by the Columbia University Institutional Review Board.

## Author Contributions

JC and EH designed the studies collaboratively. JC analyzed the data and drafted the manuscript. EH provided editorial support and they collaboratively created the current version of the manuscript.

## Conflict of Interest

The authors declare that the research was conducted in the absence of any commercial or financial relationships that could be construed as a potential conflict of interest.

## Footnotes

1 In a simple regression of the mean level of intensity onto personal sense of control, the effect size was η^2^ = 0.11. Prior to performing this and all subsequent simple regressions determining effect size, the intensity levels from all scenarios were standardized and then averaged.

2 In a simple regression of the mean level of intensity onto personal sense of control, the effect size was η^2^ = 0.11.

3 In a simple regression of the mean level of intensity onto personal sense of control, the effect size was η^2^ = 0.08.

4 In a simple regression of the mean level of intensity onto personal agency, the effect size was η^2^ = 0.31.

5 In a simple regression of the mean level of intensity onto personal agency, the effect size was η^2^ = 0.45.

6 In a simple regression of the mean level of intensity onto personal agency, the effect size was η^2^ = 0.23.

7 In a simple regression of the mean level of intensity onto personal agency, the effect size was η^2^ = 0.03.

8 The effect was still significant when not controlling for regulatory focus (*b* = 0.31, *SE* = 0.15, *z* = 2.02, *p* = 0.04, 95% CI = [0.009, 0.613]). The effect size in a simple regression with the mean of intensity regressed onto the manipulation was η^2^ = 0.06 controlling for regulatory focus, and η^2^ = 0.04 when not controlling for regulatory focus.

## Appendix A

### Scenario 1

A man’s wife is very ill with a rare cancer. There was one drug that the doctors thought might be able to save her. The drug was a form of radium developed by a druggist in the same town. The drug was very expensive to make, and the druggist only has enough for one person so far, so he is charging a premium for it. The husband worked as hard as he could to collect money from friends in the area, but was not able to raise enough to afford the drug. So he became desperate and broke into the druggist’s laboratory to steal the drug and save his wife.

### Scenario 2

A doctor has recently diagnosed a man with a rare and deadly disease. Though he won’t experience any pain or suffering, it is clear that the man will die within 6–9 days. Somehow, the girlfriend of this man heard the diagnosis before him, and begged the doctor not to tell him. She explains that her boyfriend has always wanted to visit Africa and they’re planning to leave on that trip together, and that this news would completely ruin his experience. Since he is going to die anyway, she asks the doctor to wait until after the trip. However, hospital rules and regulations dictate that the doctor must report every diagnosis to the patients as soon as they’re able to, and so the doctor does tell the man despite his girlfriend’s objections.

### Scenario 3

A woman finds herself under scrutiny for her outspoken support of religious beliefs that her co-workers find offensive. She is asked to meet with the company executive where she is told that if she publicly recants her beliefs and writes a letter of apology to her fellow employees, then she can keep her job, but if she insists on holding them, she will be fired without severance. The woman refuses the offer and loses her job instead of giving up her beliefs.

### Scenario 4

A young teacher is working at a private elementary school. The school has one zero-tolerance rule for their students: no biting. At recess one student, Elliot, approaches her claiming that he has been bitten by another student, Sarah. Upon being confronted by the teacher asking why she bit Elliot, Sarah simply replied, “I don’t know.” The teacher then said, “Now you know what happens when you bite another student, right?” Upon hearing this, Sarah turned to Elliot and said, “I’m sorry, Elliot.” Though she finds this apology touching, and doesn’t herself agree with the school’s policy, she decides to follow the school’s rules, “No. There is a zero-tolerance rule for biting, so you need to be sent home.” She sends her student, Sarah, home, according to the school’s policy.

### Scenario 5

A young man has made dinner plans with an acquaintance who doesn’t have many friends and who is undergoing hard times and needs to talk to someone. He then receives an invitation from his best friend to a party that same evening. Upon telling his friend that he already has plans, he is informed that the girl he’s been really interested in asking out will also be at this same party. Even so, he tells his friend thank you for the invitation, but that he’s sticking to the plans he has already made. He does say that he will try to come to the party afterward and so may make the meeting with his acquaintance a bit more brief.

### Scenario 6

A student is taking an exam for which he did not study. He repeatedly cheats by copying answers off of the girl sitting next to him, who he knows studied for the exam thoroughly. He doesn’t score as well as she does, but he does much better than he would have had he not cheated off her exam.

### Scenario 7

A man and his wife are walking home through the city one night when they are mugged by a pair of thugs, one armed with a handgun. The man is struck temporarily unconscious. When he comes to his senses, the two attackers appear to have beaten his wife. The armed thug has left his handgun on the ground beside him, within the reach of the husband, not knowing that the husband was alive and had regained consciousness. In a rage, the husband seizes the gun and aims it at the two attackers, threatening to kill them. Even though he is furious at the unspeakable violence the pair did to him and his wife, he decides not to kill the two attackers and instead waits until the police arrive. He and his wife then go to the hospital.

### Scenario 8

An executive decides to give one of his employees a raise. Rather than assess job performance, he decides loyalty is more important, and gives the raise to one of his friends whom he hired a few months ago. In doing so he passes over a number of other more competent employees that have been with the company much longer than his friend.

### Scenario 9

While in her bathroom, a woman realizes that the toilet is dirty and needs to be cleaned. Not having a rag handy, she takes the family American flag, tears it in half, and uses a section of it to scrub the mess off the toilet very thoroughly. After the toilet is clean she throws both sections of the flag into the garbage.

### Scenario 10

A family has had a dog for almost 12 years and has become very attached to it. One day the dog is playing in the street, gets hit by a truck and is killed. The family decides to bring home the dog’s corpse, cook it, and eat it. They bury the remains in the backyard.

### Scenario 11

A brother and sister are alone in the house and decide to make love just once. The sister is already taking birth control pills and the brother uses a condom. They both enjoy the act but decide not to do it again. They promise each other to keep it a secret.

### Scenario 12

A student at an urban college sacrifices her time on Tuesday evenings to tutor, in English and math, at-risk teenagers who are doing poorly in school and who live in a dangerous neighborhood. For doing so, she receives community service credit toward her degree.

### Scenario 13

A married couple that has just discovered that they are infertile makes the decision to adopt a child. Rather than attempting to adopt a baby, they decide to adopt a severely handicapped young girl. For doing so, they receive a substantial tax break from the federal government.

### Scenario 14

While walking through the park, a young man comes across the remains of garbage from a picnic, and sees the family leaving that left it behind. Rather than walking past it, he stops to pick it up, and disposes of it in a nearby trashcan. He then chases down the family and lectures them about the importance of taking care of the park.
